# Development of the Health Promoting Sports Club - National Audit Tool

**DOI:** 10.1093/eurpub/ckac129.271

**Published:** 2022-10-25

**Authors:** A Van Hoye, A Vuillemin, A Lane, K Dowd, S Geidne, S Kokko, A Donaldson, J Seghers, S Whiting, S Johnson

**Affiliations:** 1 Physical Activity for Health Research Cluster, University of Limerick, Limerick, Ireland; 2 LAHMESS, Université Côte d'Azur, Nice, France; 3 SHE Research Group, Technological University of the Shannon, Athlone, Ireland; 4 Örebro University, Örebro, Sweden; 5 Faculty of Sport and Health Sciences, University of Jyväskylä, Jyväskylä, Finland; 6 Centre for Sport and Social Impact, La Trobe University, Melbourne, Australia; 7 Physical Activity, Sports & Health Research Group, KU Leuven, Leuven, Belgium; 8 WHO European Office for Prevention and Control, Moscow, Russia

## Abstract

**Background:**

Sports clubs have requested support from national governing authorities to invest in health promotion (HP), by developing policies, guidelines and dedicated funding. This manuscript outlines the development of a national audit tool to review policies development and implementation to support HP in sports clubs.

**Methods:**

A 5-step process was undertaken by an international project team: (1) a rapid literature review to identify items assessing policies in physical activity, HP and sports, (2) a thematic analysis to categorize items, (3) a Delphi method to analyze item relevance, country specificity, reformulation, validation and organization, (4) face validity through an online survey and in-depth interviews with expert representatives on physical activity and sports and (5) audit tool finalization though project team consensus.

**Results:**

Eight sources were reviewed with 269 items identified. Items were coded into 25 categories with three broad themes: policies, actors and settings-based approach. The Delphi study extracted and refined 50 items and categorized them into 10 sections. After revisions from 22 surveys and 8 interviews, consensus was reached by the international project team on 41 items categorized into 11 sections: Role of ministry or department; Policies; Communication; Implementation & Dissemination; Evaluation & Measurement methods; Sub-national level policies; Funding & Coordination; Participative approach; Actors & Stakeholders; National sporting events; Case studies and Implicated stakeholders.

**Conclusions:**

To progress HP in the sports club context it is necessary to understand existing national level policies. This national audit tool will aid in monitoring and assessing national policies for health promoting sports clubs.

